# Palladium-Catalyzed Synthesis of Natural and Unnatural 2-, 5-, and 7-Oxygenated Carbazole Alkaloids from *N*-Arylcyclohexane Enaminones

**DOI:** 10.3390/molecules180910334

**Published:** 2013-08-26

**Authors:** Rafael Bautista, Pablo A. Montoya, Araceli Rebollar, Eleuterio Burgueño, Joaquín Tamariz

**Affiliations:** Departamento de Química Orgánica, Escuela Nacional de Ciencias Biológicas, Instituto Politécnico Nacional. Prol. Carpio y Plan de Ayala, México D.F. 11340, Mexico

**Keywords:** 2-oxygenated carbazoles, enaminones, palladium(II) cyclization, clausine V, glycoborine

## Abstract

A palladium-catalyzed synthesis of the carbazole framework is described, including the preparation of 2-, 5-, and 7-oxygenated natural and unnatural carbazole alkaloids. A series of *N*-arylcyclohexane enaminones, generated by condensation of cyclohexane-1,3-dione with diverse anilines, were aromatized by a Pd(0)-catalyzed thermal treatment to afford the corresponding diarylamines. The latter were submitted to a Pd(II)-catalyzed cyclization and methylation processes to provide the desired carbazoles, including clausine V. Following an inverse strategy, a new and short total synthesis of glycoborine is also reported.

## 1. Introduction

**Figure 1:**
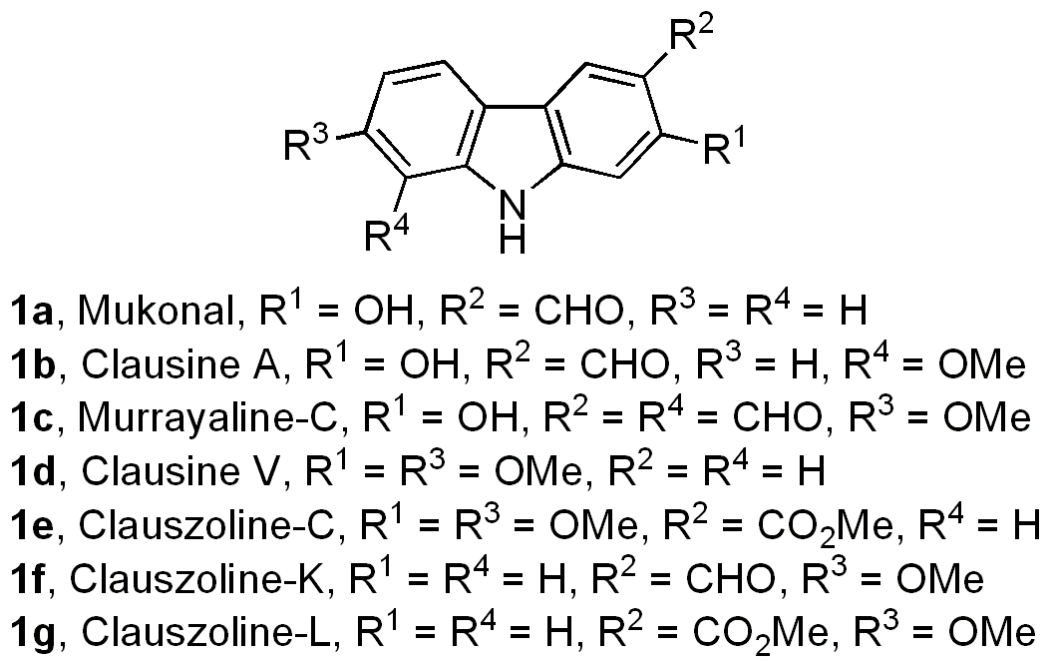
Examples of naturally occurring 2-, 7-, and 2,7-oxygenated tricyclic carbazoles.

Biologically active carbazole alkaloids, a family of natural products with a variety of molecular structures, are isolated from higher order plants of the genera *Clausena*, *Glycosmis*, *Micromelum,* and *Murraya* (Rutaceae), among other sources [1,2,3,4,5]. Specifically, a great number of 2-, 5-, 6-, 7-mono- and bis-oxygenated tricyclic carbazoles isolated from these genera [1,2,3] exhibit a broad range of significant biological activities, including compounds with anti-tumor [6,7], antiplatelet aggregative [8], antibiotic [6,9,10], anti-viral [11,12,13], anti-plasmodial [14], anti-convulsant [15], and sigma receptor antagonist [16,17] properties. Carbazole derivatives **1a**–**g** are examples of these natural alkaloids [8,18,19,20,21,22,23] (Figure 1).

In spite of the large number of 2-, 5-, 6-, 7-mono- and bis-oxygenated tricyclic natural carbazoles that have been isolated, the wide range of functional groups and substitution patterns that exists among these compounds, and their important pharmacological activity, only recently a considerable number of synthetic approaches for their efficient preparation have been published [1,2,3,24,25,26,27,28,29,30,31,32,33,34,35,36].

**Scheme 1:**
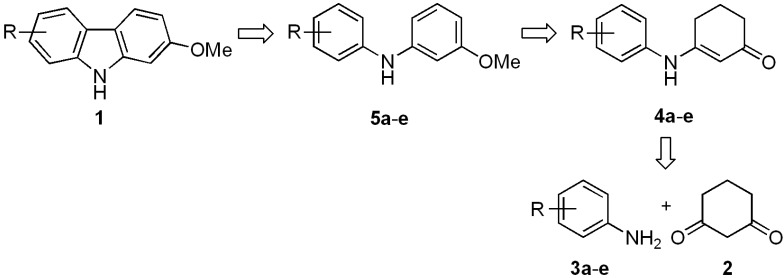
Synthetic approach for the preparation of 2-oxygenated tricyclic carbazoles **1**.

We recently described a general synthetic approach for the construction of 1-methoxycarbazoles, including the naturally occurring alkaloid glycozolicine, which was accomplished with high overall yields through a three-step reaction sequence [37]. Based on this approach, we describe herein a new synthetic route for the preparation of 2-, 7-, and 2,7-oxygenated carbazoles **1**. Starting from cyclohexene-1,3-dione (**2**) and the respective anilines **3a**–**e**, enaminones **4a**–**e** were prepared (Scheme 1). The latter were converted into diarylamines **5a**–**e** and then cyclized to the desired carbazoles **1**, via an efficient Pd-catalyzed aromatization and cyclization sequence of reactions.

## 2. Results and Discussion

### 2.1. Synthesis of Diarylamines

**Scheme 2:**
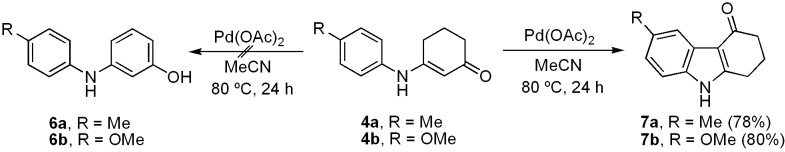
Pd(II)-catalyzed treatment of 3-anilino-2-cyclohexen-1-ones **4a**–**b**.

The catalyst-free condensation of cyclohexane-1,3-dione (**2**) with anilines **3a**–**e** provided 3-anilino-2-cyclohexen-1-ones **4a**–**e** in high yields (Table 1). However, the use of deactivated anilines, such as 3-nitro- and 4-nitroanilines, failed to provide the desired enaminones, thus limiting this procedure to anilines substituted with electron-donating groups. Applying our previous procedure for aromatization using Pd(OAc)_2_ (30% mol) [37], derivatives **4a**–**b** did not lead to the desired diarylamines **6a**–**b**, but instead furnished the carbazole frame compounds **7a**–**b** in good yields (Scheme 2). Similar results via Pd-mediated procedures have been reported for analogous substrates [1,38,39,40,41], which in turn have been transformed into the 4-oxygenated carbazoles [42]. When using other catalysts, such as mercuric acetate [43] and 2,3-dichloro-5,6-dicyano-*p*-benzoquinone (DDQ) [44,45], diarylamines **6** were indeed produced, but in very low yields (15%–20%).

**Table 1:** Scope of the reaction between cyclohexane-1,3-dione (**2**) and anilines **3a**–**e** *^a^*. 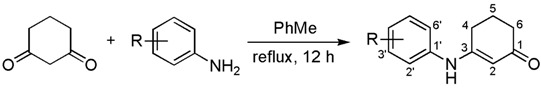

Entry	3 (Ar)	4 (%) *^b^*
1	**3a** (C_6_H_4_-4-Me)	**4a** (92)
2	**3b** (C_6_H_4_-4-OMe)	**4b** (95)
3	**3c** (C_6_H_4_-3-Me)	**4c** (90)
4	**3d** (C_6_H_4_-3-OMe)	**4d** (93)
5	**3e** (C_6_H_3_-3,5-(OMe)_2_	**4e** (96)

*^a^* Standard conditions: **2** (3.57 mmol), **3** (3.57 mmol), toluene (150 mL), reflux, 12 h.

*^b^* Isolated yields.

Due to the fact that the insertion of the aryl and cyclohexenone rings takes place via a Pd(II)-catalyzed pathway [1,2,38,39,40,41,46], we chose a Pd(0)-mediated method for carrying out such an aromatization. Initially, when **4b** was treated with Pd/C (5%) at different concentrations (1–6 mol%) with MeOH as the solvent and heating to 50–200 °C in a sealed vessel, diarylamine **6b** was not obtained and the starting material was recovered. However, the desired transformation was achieved by increasing both the palladium(0) loading on charcoal (10%) (1.9%–5.7% mol) and the reaction temperature (Table 2, entries 1–3). The use of the Pd(0)-mediated aromatization method for similar substrates or carbazole derivatives in moderate to good yields has been reported [42,47,48,49,50,51,52]. Reagents such as DDQ [44,45,53] and chloranil [54] have also been successfully applied to achieve analogous conversions [43,55].

Although the preparation and purification of diarylamines **6a**–**b** and **6d**–**e** resulted in high yields (Table 2, entries 3–4 and 6–7), the relative instability of these compounds under the conditions of the following cyclization reaction made it necessary to protect the phenol moiety. In order to achieve this protection and taking into account that there are many naturally occurring methoxy-containing oxygenated carbazoles, we decided to obtain the methylated derivatives **5a**–**e**. For this purpose, we employed a direct sequential procedure for the dehydrogenation and methylation of phenols **6** without purification (Table 2, entries 3–7). Thus, the series of compounds **5a**–**e** was prepared in high yields (81%–87%).

**Table 2:** Conversion of 3-anilino-2-cyclohexen-1-ones **4a**–**e** into diarylamines **6a**–**b**, **6d**–**e** and **5a**–**e** *^a^*. 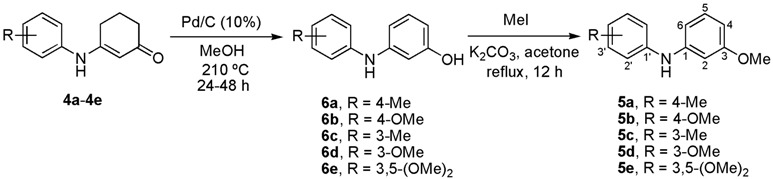

Entry	4 (R)	Pd/C (10%) (mol%) *^b^*	6 (%) *^c^*	5 (%) *^d^*
1 *^e^*	**4b** (4-OMe)	1.9	**6b** (65)	----
2 *^f^*	**4b** (4-OMe)	3.8	**6b** (75)	----
3	**4b** (4-OMe)	5.7	**6b** (87)	**5b** (85)
4	**4a** (4-Me)	5.7	**6a** (85)	**5a** (83)
5	**4c** (3-Me)	5.7	(g)	**5c** (87)
6	**4d** (3-OMe)	5.7	**6d** (84)	**5d** (81)
7	**4e** (3,5-(OMe)_2_	5.7	**6e** (88)	**5e** (86)

*^a^* Standard conditions: (a) Preparation of diarylamines **6**: **4** (0.81–1.00 mmol), Pd/C (10%), MeOH, 210 °C, 48 h; (b) Preparation of diarylamines **5a**–**e**: Aromatization step: **4** (0.82–1.00 mmol), Pd/C (10%), MeOH, 210 °C, 24 h; Methylation step: **6** (1.0 mol equiv.), MeI (2.0 mol equiv.), K_2_CO_3_ (1.5 mol equiv.), acetone, reflux, 12 h. *^b^* Calculated for Pd(0). *^c^* Isolated yields. *^d^* Isolated yields for the two steps. *^e^* At 180 °C for 12 h. *^f^* At 200 °C for 48 h. *^g^* Not isolated.

### 2.2. Synthesis of Carbazoles

The final cyclization step of diarylamines **5a**–**e** was successfully carried out by following the protocol originally developed by Knölker and coworkers [40,56,57], later applied by others [28,29], and optimized in our syntheses of natural carbazoles [37,44]. Thus, the conversion of the series **5a**–**c** and **5e** into the carbazole derivatives **1h**–**k** resulted in good yields (80%–92%) (Table 3). It is noteworthy that the cyclization of **5d** provided clausine V (**1d**) in high yield (90%) [22,33].

**Scheme 3:**
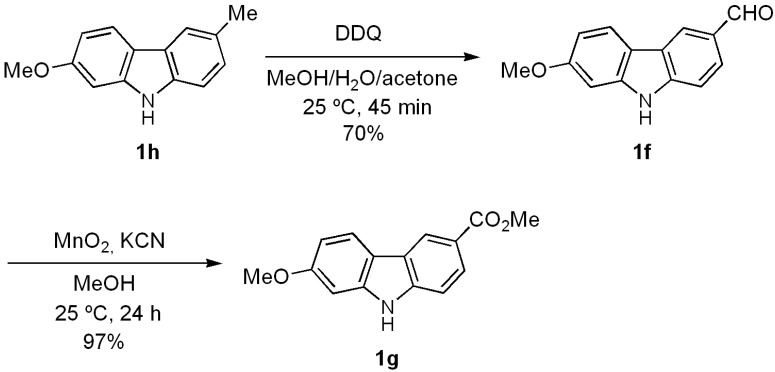
Preparation of natural carbazoles clauszoline-K (**1f**) and clauszoline-L (**1g**).

With the aim of testing the utility of this methodology for the total synthesis of natural 7-oxygenated tricyclic carbazoles, we carried out the conversion of derivative **1h** into clauszoline-K (**1f**) and clauszoline-L (clausine C, **1g**). Thus, upon applying the well-known procedure [58,59] for the synthesis of these [32] and other natural carbazoles [44], carbazole **1h** was treated with DDQ in a mixture of MeOH/H_2_O/acetone (1:1:1) at room temperature for 45 min to give **1f** in 70% yield (Scheme 3).

**Table 3:** Preparation of carbazoles **1d** and **1h**–**k** via Pd(II)-catalyzed cyclization of diarylamines **5a**–**e** *^a^*. 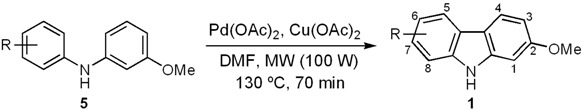

Entry	5 (R)	1	Isolated yield (%) ^b^
1	**5a** (4-Me)	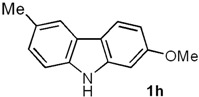	80
2	**5b** (4-OMe)	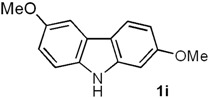	87
3	**5c** (3-Me)	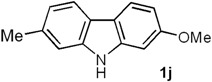	82
4	**5d** (3-OMe)	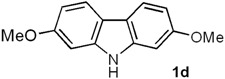	90
5	**5e** (3,5-(OMe)2	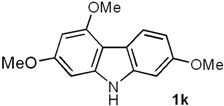	92

*^a^* Standard conditions: **5** (0.32 –0.47 mmol), Pd(OAc)_2_ (10 mol%), Cu(OAc)_2_ (2.5 mol equiv.), DMF, MW (100 W), 130 °C, 70 min. *^b^* Isolated yields.

The latter was oxidized with a mixture of MnO_2_/KCN in MeOH [58] to furnish clauszoline-L (**1g**) in almost quantitative yield. The spectral data of the products obtained agree with those described for the natural [20,23] and synthetic [58] products.

### 2.3. Total Synthesis of Glycoborine (Glycrophylamine, **9**)

**Scheme 4:**
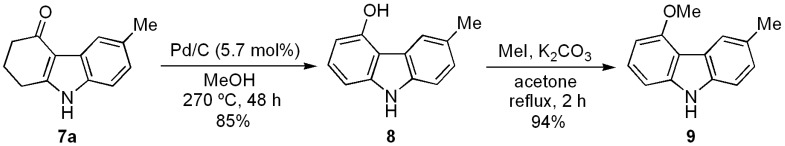
Preparation of natural carbazole glycoborine (**9**).

Recently, 5-methoxy-3-methylcarbazole (**9**) was isolated from the roots and branches of *Glycosmis macrophylla* and named glycrophylamine. This compound showed cytotoxic activity against NC1-H187 cancerigene cells [60]. However, the same carbazole had been isolated from *Glycosmis arborea* a decade earlier, and named glycoborine. This was the first 5-oxygenated tricyclic natural carbazole ever isolated [61]. Nowadays, three routes of synthesis have been developed for **9** based on Fischer [61], Japp-Klingemann [62], and Cadogan cyclizations [33] as the key step. We herein describe a new total synthesis of **9** starting from the key precursor tetrahydrocarbazole **7a** (Scheme 4), which was efficiently prepared from **4a** (Scheme 2).

When a mixture of **7a**, Pd/C (10%) (5.7% mol) and anhydrous MeOH was heated in a sealed vessel to 270 °C for 48 h, 5-hydroxy-3-methylcarbazole (**8**) was isolated and then purified in good yield (Scheme 4). Methylation of the latter under the usual reaction conditions provided the desired natural carbazole **9**, which was synthesized in four steps with high overall yield (53%). The spectral data of **9** agree with those described for the natural [60,61] and synthetic [33,62] products.

All the structures of intermediates and products described in these synthetic sequences were characterized by ^1^H- and ^13^C-NMR spectroscopy, with the help of 2D (HMQC and HMBC) experiments and mass spectrometric techniques (MS and HRMS).

## 3. Experimental

### 3.1. General

Melting points (uncorrected) were determined with an Electrothermal capillary melting point apparatus. IR spectra were recorded on a Perkin-Elmer 2000 spectrophotometer. ^1^H (300 or 500 MHz) and ^13^C-NMR (75 or 125 MHz) spectra were recorded on Varian Mercury-300 or Varian VNMR System instruments, with TMS as internal standard. Mass spectra (MS) and high-resolution mass spectra (HRMS) were obtained, in electron impact (EI) (70 eV) mode, on Thermo-Finnigan Polaris Q and Jeol JSM-GcMateII spectrometers, respectively. Microwave (MW) irradiation was performed on a CEM MW reactor. Analytical thin-layer chromatography was carried out using E. Merck silica gel 60 F_254_ coated 0.25 plates, visualized by a long- and short-wavelength UV lamp. Flash column chromatography was performed over Natland International Co. silica gel (230–400 mesh). All air moisture sensitive reactions were carried out under nitrogen using oven-dried glassware. Toluene, MeOH, and MeCN were freshly distilled over sodium and DMF over calcium hydride prior to use. Acetone was dried by distillation after treatment with potassium permanganate. K_2_CO_3_ was dried overnight at 200 °C prior to use. All other reagents were used without further purification.

### 3.2. Synthesis and Characterization

*3-(p-Tolylamino)cyclohex-2-en-1-one* (**4a**) [63]. In a 250 mL, three necked, round-bottomed flask equipped with a magnetic stirring bar, rubber septum, a water condenser and a Dean-Stark trap, under N_2_ atmosphere, a mixture of **2** (0.400 g, 3.57 mmol) and **3a** (0.382 g, 3.37 mmol) in dry toluene (150 mL) was stirred at reflux for 12 h. The solvent was removed under vacuum, and the residue purified by column chromatography over silica gel (10 g/g of crude, hexane/EtOAc, 1:1) to give **4a** (0.66 g, 92%) as a pale yellow solid. *R*_f_ 0.15 (hexane/EtOAc, 1:1); mp 248–249 °C. IR (KBr): ν_max_ 3214, 3029, 2937, 1573, 1512, 1361, 1311, 1245, 1183, 1141, 818 cm^−1^. ^1^H-NMR (500 MHz, CDCl_3_): δ = 1.98 (qu, *J* = 6.5 Hz, 2H, H-5), 2.31 (br t, *J* = 6.5 Hz, 2H, H-6), 2.32 (s, 3H, C*H*_3_), 2.48 (t, *J* = 6.5 Hz, 2H, H-4), 5.48 (s, 1H, H-2), 6.99–7.03 (m, 2H, H-2′), 7.07–7.11 (m, 2H, H-3′), 7.12 (br s, 1H, NH). ^13^C-NMR (125 MHz, CDCl_3_): δ = 20.9 (*C*H_3_), 21.8 (C-5), 29.5 (C-4), 36.4 (C-6), 99.0 (C-2), 124.0 (C-2′), 129.7 (C-3′), 135.3 (C-4′), 135.5 (C-1′), 163.1 (C-3), 198.1 (C-1). MS (70 eV): *m/z* (%) 201 (M^+^, 74), 184 (26), 173 (100), 144 (53), 130 (29), 106 (13), 91 (12), 77 (10). HRMS (EI): *m/z* [M^+^] calcd for C_13_H_15_NO: 201.1154; found: 201.1156.

*3-(4-Methoxyphenylamino)cyclohex-2-en-1-one* (**4b**). Following the procedure described for **4a**, using **2** (0.400 g, 3.57 mmol) and **3b** (0.439 g, 3.57 mmol), **4b** (0.74 g, 95%) was obtained as a pale yellow solid. *R*_f_ 0.12 (hexane/EtOAc, 1:1); mp 166–167 °C [Lit. [64] 164–166 °C]. IR (KBr): ν_max_ 3218, 3039, 2946, 1513, 1412, 1365, 1243, 1180, 1135, 1032, 834, 716 cm^−1^. ^1^H-NMR (500 MHz, CDCl_3_): δ = 1.97 (qu, *J* = 6.5 Hz, 2H, H-5), 2.30 (t, *J* = 6.5 Hz, 2H, H-6), 2.47 (t, *J* = 6.5 Hz, 2H, H-4), 3.78 (s, 3H, C*H*_3_O), 5.34 (s, 1H, H-2), 6.80–6.84 (m, 2H, H-3′), 7.02–7.06 (m, 2H, H-2′), 7.13 (br s, 1H, NH). ^13^C-NMR (125 MHz, CDCl_3_): δ = 21.8 (C-5), 29.3 (C-4), 36.4 (C-6), 55.4 (*C*H_3_O), 98.6 (C-2), 114.4 (C-3′), 126.1 (C-2′), 130.8 (C-1′), 157.5 (C-4′), 164.0 (C-3), 198.0 (C-1). MS (70 eV): *m/z* (%) 217 (M^+^, 100), 200 (55), 189 (43), 174 (20), 160 (98), 146 (30), 130 (23), 117 (12), 77 (10). HRMS (EI): *m/z* [M^+^] calcd for C_13_H_15_NO_2_: 217.1103; found: 217.1110.

*3-(m-Tolylamino)cyclohex-2-en-1-one* (**4c**). Following the procedure described for **4a**, **4c** (0.65 g, 90%) was obtained as a pale yellow oil from **2** (0.400 g, 3.57 mmol) and **3c** (0.382 g, 3.57 mmol). *R*_f_ 0.15 (hexane/EtOAc, 1:1). IR (film): ν_max_ 3256, 3066, 2959, 1546, 1453, 1360, 1244, 1185, 1136, 829, 795, 728 cm^−1^. ^1^H-NMR (500 MHz, CDCl_3_): δ = 1.98 (qu, *J* = 6.5 Hz, 2H, H-5), 2.29 (s, 3H, C*H*_3_), 2.32 (t, *J* = 6.5 Hz, 2H, H-6), 2.49 (t, *J* = 6.5 Hz, 2H, H-4), 5.55 (s, 1H, H-2), 6.91-6.95 (m, 2H, H-4′, H-6′), 6.96 (br s, 1H, H-2′), 7.17 (t, *J* = 8.0 Hz, 1H, H-5′), 7.18 (br s, 1H, NH). ^13^C-NMR (125 MHz, CDCl_3_): δ 21.3 (*C*H_3_), 21.8 (C-5), 29.6 (C-4), 36.4 (C-6), 99.3 (C-2), 120.9 (C-6′), 124.4 (C-2′), 126.2 (C-4′), 129.0 (C-5′), 138.0 (C-3′), 139.1 (C-1′), 162.9 (C-3), 198.3 (C-1). MS (70 eV): *m/z* (%) 201 (M^+^, 79), 184 (49), 173 (100), 158 (16), 144 (88), 130 (42), 106 (13), 91 (19), 77 (16). HRMS (EI): *m/z* [M^+^] calcd for C_13_H_15_NO: 201.1154; found: 201.1160.

*3-(3-Methoxyphenylamino)cyclohex-2-en-1-one* (**4d**). Following the procedure described for **4a**, using **2** (0.400 g, 3.57 mmol) and **3d** (0.439 g, 3.57 mmol), **4d** (0.72 g, 93%) was obtained as a pale yellow solid. *R*_f_ 0.11 (hexane/EtOAc, 1:1); mp 126–127 °C [Lit. [64] 122.5–124 °C; [65] 126–128 °C]. IR (KBr): ν_max_ 3278, 3196, 3133, 2937, 1540, 1425, 1357, 1318, 1245, 1191, 1143, 1051, 871, 733 cm^−1^. ^1^H-NMR (500 MHz, CDCl_3_): δ 1.98 (qu, *J* = 6.5 Hz, 2H, H-5), 2.32 (t, *J* = 6.5 Hz, 2H, H-6), 2.50 (t, *J* = 6.5 Hz, 2H, H-4), 3.75 (s, 3H, C*H*_3_O), 5.59 (s, 1H, H-2), 6.66–6.70 (m, 2H, H-2′, H-6′), 6.72 (dm, *J* = 8.0 Hz, 1H, H-4′), 7.16–7.21 (m, 1H, H-5′), 7.29 (br s, 1H, NH). ^13^C-NMR (125 MHz, CDCl_3_): δ 21.8 (C-5), 29.5 (C-4), 36.4 (C-6), 55.2 (*C*H_3_O), 99.6 (C-2), 109.7 (C-2′), 110.8 (C-6′), 116.0 (C-4′), 129.9 (C-5′), 139.4 (C-1′), 160.2 (C-3′), 162.7 (C-3), 198.4 (C-1). MS (70 eV): *m/z* (%) 217 (M^+^, 99), 200 (59), 189 (46), 160 (100), 146 (32), 130 (23), 117 (11), 77 (7). HRMS (EI): *m/z* [M^+^] calcd for C_13_H_15_NO_2_: 217.1103; found: 217.1101.

*3-(3,5-Dimethoxyphenylamino)cyclohex-2-en-1-one* (**4e**). Following the procedure described for **4a**, with **2** (0.400 g, 3.57 mmol) and **3e** (0.546 g, 3.57 mmol), **4e** (0.85 g, 96%) was obtained as a pale yellow solid. *R*_f_ 0.12 (hexane/EtOAc, 1:1); mp 139–140 °C. IR (KBr): ν_max_ 3272, 2940, 1598, 1582, 1538, 1462, 1423, 1361, 1253, 1186, 1153, 1055, 824 cm^−1^. ^1^H-NMR (500 MHz, CDCl_3_): δ = 1.99 (qu, *J* = 6.5 Hz, 2H, H-5), 2.33 (t, *J* = 6.5 Hz, 2H, H-6), 2.49 (t, *J* = 6.5 Hz, 2H, H-4), 3.73 (s, 6H, 2C*H*_3_O), 5.64 (s, 1H, H-2), 6.24 (t, *J* = 2.0 Hz, 1H, H-4′), 6.30 (d, *J* = 2.0 Hz, 2H, H-2′, H-6′), 7.06 (br s, 1H, NH). ^13^C-NMR (125 MHz, CDCl_3_): δ = 21.8 (C-5), 29.6 (C-4), 36.4 (C-6), 55.3 (*C*H_3_O), 97.4 (C-4′), 100.2 (C-2), 102.1 (C-2′, C-6′), 139.9 (C-1′), 161.2 (C-3′, C-5′), 162.3 (C-3), 198.3 (C-1). MS (70 eV): *m/z* (%) 247 (M^+^, 37), 230 (100), 219 (25), 190 (83), 160 (18), 135 (30), 120 (14), 77 (7). HRMS (EI): *m/z* [M^+^] calcd for C_14_H_17_NO_3_: 247.1208; found: 247.1207.

*3-(p-Tolylamino)phenol* (**6a**). In a threaded ACE glass pressure tube with a sealed Teflon screw cap, under N_2_ atmosphere, a mixture of **4a** (0.20 g, 1.0 mmol) and Pd/C (10%) (0.060 g, 0.057 mmol) in dry MeOH (2.5 mL) was stirred at 210 °C for 48 h. The solvent was removed under vacuum, and the residue purified by column chromatography over silica gel (20 g/g of crude, hexane/EtOAc, 80:20), to give **6a** (0.168 g, 85%) as a pale grey solid. *R*_f_ 0.55 (hexane/EtOAc, 7:3); mp 81–82 °C [Lit. [66] 82 °C]. IR (film): ν_max_ 3394, 1606, 1512, 1493, 1332, 1243, 1155, 969, 815, 766 cm^−1^. ^1^H-NMR (300 MHz, CDCl_3_): δ = 2.27 (s, 3H, C*H*_3_), 5.58 (br s, 1H, NH), 6.31 (dd, *J* = 7.8, 2.1 Hz, 1H, H-4), 6.44 (t, *J* = 2.1 Hz, 1H, H-2), 6.51 (dd, *J* = 7.8, 2.1 Hz, 1H, H-6), 6.92–6.99 (m, 2H, H-2′), 6.99–7.07 (m, 3H, H-3′, H-5). ^13^C-NMR (75.4 MHz, CDCl_3_): δ = 20.6 (*C*H_3_), 103.2 (C-2), 107.1 (C-4), 108.9 (C-6), 119.5 (C-2′), 129.8 (C-3′), 130.2 (C-5), 131.2 (C-4′), 139.7 (C-1′), 145.6 (C-1), 156.6 (C-3). MS (70 eV): *m/z* (%) 199 (M^+^, 100), 183 (19), 170 (22), 154 (35), 128 (14), 91 (83), 65 (18). HRMS (EI): *m/z* [M^+^] calcd for C_13_H_13_NO: 199.0997; found: 199.0998.

*3-(4-Methoxyphenylamino)phenol* (**6b**). Following the procedure described for **6a**, with **4b** (0.200 g, 0.92 mmol) and Pd/C (10%) (0.060 g, 0.057 mmol), **6b** (0.172 g, 87%) was obtained as a pale grey solid. *R*_f_ 0.50 (hexane/EtOAc, 8:2); mp 66–67 °C [Lit. [66] 67–68 °C]. IR (KBr): ν_max_ 3379, 1601, 1526, 1504, 1459, 1291, 1239, 1174, 1110, 1027, 735 cm^−1^. ^1^H-NMR (300 MHz, CDCl_3_): δ = 3.79 (s, 3H, C*H*_3_O), 6.50 (br s, 1H, NH), 6.28 (ddd, *J* = 8.0, 2.4, 0.6 Hz, 1H, H-4), 6.37 (t, *J* = 2.4 Hz, 1H, H-2), 6.44 (ddd, *J* = 8.0, 2.4, 0.6 Hz, 1H, H-6), 6.82–6.89 (m, 2H, H-3′), 7.04 (t, *J* = 8.0 Hz, 1H, H-5), 7.04–7.10 (m, 2H, H-2′). ^13^C-NMR (75.4 MHz, CDCl_3_): δ = 55.5 (*C*H_3_O), 101.9 (C-2), 106.3 (C-4), 108.0 (C-6), 114.6 (C-3′), 122.9 (C-2′), 130.3 (C-5), 135.1 (C-1′), 146.9 (C-1), 155.4 (C-4′), 156.7 (C-3). MS (70 eV): *m/z* (%) 215 (M^+^, 100), 201 (6), 185 (7), 172 (5), 146 (4), 132 (5), 91 (11). HRMS (EI): *m/z* [M^+^] calcd for C_13_H_13_NO_2_: 215.0946; found: 215.0952.

*3-(3-Methoxyphenylamino)phenol* (**6d**) [66]. Following the procedure described for **6a**, with **4d** (0.200 g, 0.92 mmol) and Pd/C (10%) (0.060 g, 0.057 mmol), **6d** (0.166 g, 84%) was obtained as a purple oil. *R*_f_ 0.51 (hexane/EtOAc, 8:2). IR (film): ν_max_ 3411, 1645, 1489, 1156, 764 cm^−1^. ^1^H-NMR (300 MHz, CDCl_3_): δ = 3.71 (s, 3H, C*H*_3_O), 5.76 (br s, 1H, NH), 6.38 (ddd, *J* = 8.1, 2.4, 0.9 Hz, 1H, H-4), 6.46 (dm, *J* = 7.8 Hz, 1H, H-4′), 6.54 (t, *J* = 2.4 Hz, 1H, H-2), 6.56–6.65 (m, 3H, H-2′, H-6, H-6′), 7.05 (t, *J* = 8.1 Hz, 1H, H-5), 7.11 (t, *J* = 7.8 Hz, 1H, H-5′). ^13^C-NMR (75.4 MHz, CDCl_3_): δ = 55.1 (*C*H_3_O), 103.8 (C-2′), 104.6 (C-2), 106.3 (C-4′), 108.0 (C-4), 110.1 (C-6′), 110.7 (C-6), 130.0 (C-5′), 130.2 (C-5), 144.0 (C-1′), 144.3 (C-1), 156.6 (C-3), 160.3 (C-3′). MS (70 eV): *m/z* (%) 215 (M^+^, 26), 199 (21), 182 (25), 160 (31), 146 (45), 130 (25), 109 (23), 51 (100). HRMS (EI): *m/z* [M^+^] calcd for C_13_H_13_NO_2_: 215.0946; found: 215.0952.

*3-(3,5-Dimethoxyphenylamino)phenol* (**6e**). Following the procedure described for **6a**, with **4e** (0.200 g, 0.81 mmol) and Pd/C (10%) (0.060 g, 0.057 mmol), **6e** (0.174 g, 88%) was obtained as a yellow oil. *R*_f_ 0.49 (hexane/EtOAc, 8:2). IR (film): ν_max_ 3379, 2917, 1594, 1481, 1203, 1152, 1065, 821 cm^−1^. ^1^H-NMR (300 MHz, CDCl_3_): δ = 3.73 (s, 6H, 2C*H*_3_O), 5.74 (br s, 1H, NH), 6.07 (t, *J* = 2.1 Hz, 1H, H-4′), 6.23 (d, *J* = 2.1 Hz, 2H, H-2′, H-6′), 6.39 (dd, *J* = 8.1, 2.4 Hz, 1H, H-4), 6.55 (dd, *J* = 2.4, 2.1 Hz, 1H, H-2), 6.62 (ddd, *J* = 8.1, 2.1, 0.9 Hz, 1H, H-6), 7.08 (t, *J* = 8.1 Hz, 1H, H-5). ^13^C-NMR (75.4 MHz, CDCl_3_): δ = 55.3 (2*C*H_3_O), 93.2 (C-4′), 96.3 (C-2′, C-6′), 105.0 (C-2), 108.2 (C-4), 110.6 (C-6), 130.3 (C-5), 144.1 (C-1′), 144.7 (C-1), 156.6 (C-3), 161.4 (C-3′, C-5′). MS (70 eV): *m/z* (%) 245 (M^+^, 3), 154 (14), 153 (96), 125 (15), 124 (100), 94 (25), 92 (22). HRMS (EI): *m/z* [M^+^] calcd for C_14_H_15_NO_3_: 245.1052; found: 245.1059.

*3-Methoxy-N-(p-tolyl)aniline* (**5a**) [67]. In a threaded ACE glass pressure tube with a sealed Teflon screw cap, under N_2_ atmosphere, a mixture of **4a** (0.20 g, 1.0 mmol) and Pd/C (10%) (0.060 g, 0.057 mmol) in dry MeOH (2.5 mL) was stirred at 210 °C for 24 h. After removing the solvent under vacuum, K_2_CO_3_, (0.200 g, 1.45 mmol) and CH_3_I (0.281 g, 1.98 mmol) in dry acetone (20 mL) were added, and the mixture was heated to reflux for 12 h. The solvent was removed under vacuum and the residue purified by column chromatography over silica gel (20 g/g of crude, hexane/EtOAc, 90:10), to give **5a** (0.176 g, 83%) as a white solid. *R*_f_ 0.60 (hexane/EtOAc, 7:3); mp 49–50 °C. IR (KBr): ν_max_ 3367, 1598, 1493, 1462, 1256, 1157, 1032, 950, 832, 774 cm^−1^. ^1^H-NMR (500 MHz, CDCl_3_): δ = 2.30 (s, 3H, C*H*_3_), 3.75 (s, 3H, C*H*_3_O), 6.00 (br s, 1H, NH), 6.42 (ddd, *J* = 8.5, 2.5, 0.5 Hz, 1H, H-4), 6.55-6.59 (m, 2H, H-2, H-6), 6.98–7.02 (m, 2H, H-2′), 7.06-7.10 (m, 2H, H-3′), 7.12 (tm, *J* = 8.5 Hz, 1H, H-5). ^13^C-NMR (125 MHz, CDCl_3_): δ = 20.7 (*C*H_3_), 55.1 (*C*H_3_O), 102.4 (C-2), 105.5 (C-4), 109.4 (C-6), 119.4 (C-2′), 129.8 (C-3′), 130.0 (C-5), 131.2 (C-4′), 140.0 (C-1′), 145.4 (C-1), 160.7 (C-3). MS (70 eV): *m/z* (%) 213 (M^+^, 100), 200 (23), 189 (24), 174 (21), 160 (39), 130 (11), 91 (12), 84 (9). HRMS (EI): *m/z* [M^+^] calcd for C_14_H_15_NO: 213.1154; found: 213.1153.

*3-Methoxy-N-(4-methoxyphenyl)aniline* (**5b**) [29]. Following the procedure described for **5a** using **4b** (0.200 g, 0.92 mmol), Pd/C (10%) (0.055 g, 0.052 mmol), K_2_CO_3_ (0.190 g, 1.38 mmol) and MeI (0.261 g, 1.84 mmol), **5b** (0.179 g, 85%) was obtained as a white solid. *R*_f_ 0.55 (hexane/EtOAc, 7:3); mp 99–100 °C. IR (KBr): ν_max_ 3400, 1597, 1509, 1460, 1239, 1157, 1035, 825, 768 cm^−1^. ^1^H-NMR (300 MHz, CDCl_3_): δ = 3.75 (s, 3H, C*H*_3_O), 3.79 (s, 3H, C*H*_3_O), 6.51 (br s, 1H, NH), 6.38 (br dd, *J* = 7.8, 2.1 Hz, 1H, H-4), 6.44–6.51 (m, 2H, H-2, H-6), 6.83–6.89 (m, 2H, H-3′), 7.04–7.10 (m, 2H, H-2′), 7.11 (t, *J* = 7.8 Hz, 1H, H-5). ^13^C-NMR (75.4 MHz, CDCl_3_): δ = 55.1 (*C*H_3_O-C3), 55.5 (*C*H_3_O-C4′), 101.1 (C-2), 104.6 (C-4), 108.2 (C-6), 114.6 (C-3′), 122.7 (C-2′), 130.0 (C-5), 135.3 (C-1′), 146.6 (C-1), 155.4 (C-4′), 160.7 (C-3). MS (70 eV): *m/z* (%) 229 (M^+^, 90), 216 (47), 214 (100), 186 (19), 171 (21), 142 (15), 115 (21). HRMS (EI): *m/z* [M^+^] calcd for C_14_H_15_NO_2_: 229.1103; found: 229.1111.

*3-Methoxy-N-(m-tolyl)aniline* (**5c**). Following the procedure described for **5a**, with **4c** (0.20 g, 1.0 mmol), Pd/C (10%) (0.060 g, 0.057 mmol), K_2_CO_3_ (0.200 g, 1.45 mmol) and MeI (0.281 g, 1.98 mmol), **5c** (0.18 g, 87%) was obtained as a yellow oil. *R*_f_ 0.59 (hexane/EtOAc, 7:3). IR (film): ν_max_ 3391, 1589, 1490, 1266, 1203, 1155, 1042, 765, 687 cm^−1^. ^1^H-NMR (300 MHz, CDCl_3_): δ = 2.31 (s, 3H, C*H*_3_), 3.77 (s, 3H, C*H*_3_O), 5.67 (br s, 1H, NH), 6.47 (br dd, *J* = 7.8, 2.4 Hz, 1H, H-4), 6.62-6.67 (m, 2H, H-2, H-6), 6.76 (br d, *J* = 7.2 Hz, 1H, H-4′), 6.88–6.94 (m, 2H, H-2′, H-6′), 7.12–7.20 (m, 2H, H-5, H-5′). ^13^C-NMR (75.4 MHz, CDCl_3_): δ = 21.5 (*C*H_3_), 55.2 (*C*H_3_O), 103.2 (C-2), 105.9 (C-4), 110.2 (C-6), 115.4 (C-6′), 119.0 (C-2′), 122.1 (C-4′), 129.1 (C-5′), 130.0 (C-5), 139.2 (C-3′), 142.7 (C-1′), 144.6 (C-1), 160.6 (C-3). MS (70 eV): *m/z* (%) 213 (M^+^, 100), 200 (32), 189 (35), 174 (26), 160 (44), 130 (13), 92 (11), 77 (11). HRMS (EI): *m/z* [M^+^] calcd for C_14_H_15_NO: 213.1154; found: 213.1161.

*bis(3-Methoxyphenyl)amine* (**5d**) [26]. Following the procedure described for **5a**, with **4d** (0.20 g, 0.92 mmol), Pd/C (10%) (0.055 g, 0.052 mmol), K_2_CO_3_ (0.190 g, 1.38 mmol) and MeI (0.261 g, 1.84 mmol), **5d** (0.171 g, 81%) was obtained as a white solid. *R*_f_ 0.55 (hexane/EtOAc, 7:3); mp 154–155 °C. IR (film): ν_max_ 3393, 1592, 1490, 1270, 1207, 1155, 1040, 832, 760, 685 cm^−1^. ^1^H-NMR (300 MHz, CDCl_3_): δ = 3.74 (s, 6H, 2C*H*_3_O), 5.78 (br s, 1H, NH), 6.47 (ddm, *J* = 8.1, 2.4 Hz, 2H, H-4, H-4′), 6.61–6.68 (m, 4H, H-2, H-2′, H-6, H-6′), 7.14 (t, *J* = 8.1 Hz, 2H, H-5, H-5′). ^13^C-NMR (75.4 MHz, CDCl_3_): δ = 55.1 (2*C*H_3_O), 103.6 (C-2, C-2′), 106.3 (C-4, C-4′), 110.4 (C-6, C-6′), 130.0 (C-5, C-5′), 144.1 (C-1, C-1′), 160.5 (C-3, C-3′). MS (70 eV): *m/z* (%) 229 (M^+^, 100), 217 (10), 200 (12), 189 (6), 170 (11), 160 (9), 154 (12), 142 (9), 115 (5). HRMS (EI): *m/z* [M^+^] calcd for C_14_H_15_NO_2_: 229.1103; found: 229.1104.

*3,5-Dimethoxy-N-(3-methoxyphenyl)aniline* (**5e**). Following the procedure described for **5a**, with **4e** (0.200 g, 0.818 mmol), Pd/C (10%) (0.050 g, 0.047 mmol), K_2_CO_3_ (0.167 g, 1.21 mmol) and MeI (0.230 g, 1.62 mmol), **5e** (0.18 g, 86%) was obtained as a colorless oil. *R*_f_ 0.52 (hexane/EtOAc, 7:3). IR (film): ν_max_ 3735, 1590, 1541, 1457, 1203, 1150, 1057 cm^−1^. ^1^H-NMR (500 MHz, CDCl_3_): δ = 3.74 (s, 6H, 2C*H*_3_O), 3.76 (s, 3H, C*H*_3_O-3), 5.73 (br s, 1H, NH), 6.07 (t, *J* = 2.0 Hz, 1H, H-4), 6.24 (d, *J* = 2.0 Hz, 2H, H-2, H-6), 6.49 (ddd, *J* = 8.0, 2.0, 1.0 Hz, 1H, H-4′), 6.65 (t, *J* = 2.0 Hz, 1H, H-2′), 6.67 (ddd, *J* = 8.0, 2.0, 1.0 Hz, 1H, H-6′), 7.15 (t, *J* = 8.0 Hz, 1H, H-5′). ^13^C-NMR (125 MHz, CDCl_3_): δ = 55.1 (*C*H_3_O-3′), 55.2 (2*C*H_3_O), 93.3 (C-4), 96.2 (C-2, C-6), 104.2 (C-2′), 106.7 (C-4′), 111.0 (C-6′), 130.0 (C-5′), 144.0 (C-1), 144.9 (C-1′), 160.6 (C-3′), 161.6 (C-3, C-5). MS (70 eV): *m/z* (%) 259 (M^+^, 7), 257 (97), 242 (100), 214 (49), 199 (42), 184 (13), 156 (8), 128 (7). HRMS (EI): *m/z* [M^+^] calcd for C_15_H_17_NO_3_: 259.1208; found: 259.1209.

*2-Methoxy-6-methyl-9H-carbazole* (**1h**). A mixture of **5a** (0.100 g, 0.47 mmol), Pd(AcO)_2_ (0.0105 g, 0.047 mmol) and Cu(AcO)_2_ (0.211 g, 1.17 mmol) in dry DMF (0.5 mL), under N_2_ atmosphere, was stirred and heated at 130 °C for 70 min under MW irradiation (100 W). The solvent was removed under vacuum by adding toluene, and the azeotropic distillation was continued until no solvent remained. The residue was purified by column chromatography over silica gel (10 g/g of crude, hexane/EtOAc, 95:5), to give **1h** (0.079 g, 80%) as a white solid. *R*_f_ 0.60 (hexane/EtOAc, 7:3); mp 226–227 °C [Lit. [32] 227–228 °C]. IR (film): ν_max_ 3392, 1659, 1026, 826, 764, 687 cm^−1^. ^1^H-NMR (500 MHz, DMSO-*d_6_*/acetone-*d_6_*, 3:7): δ = 2.46 (s, 3H, C*H*_3_), 3.86 (s, 3H, C*H*_3_O), 6.76 (dd, *J* = 8.4, 2.4 Hz, 1H, H-3), 6.99 (d, *J* = 2.4 Hz, 1H, H-1), 7.11 (dd, *J* = 8.2, 1.5 Hz, 1H, H-7), 7.33 (d, *J* = 8.2 Hz, 1H, H-8), 7.78 (br s, 1H, H-5), 7.91 (d, *J* = 8.4 Hz, 1H, H-4), 10.6 (br s, 1H, NH). ^13^C-NMR (125 MHz, DMSO-*d_6_*/acetone-*d_6_*, 3:7): δ = 21.2 (*C*H_3_), 55.4 (*C*H_3_O), 95.0 (C-1), 108.1 (C-3), 110.8 (C-8), 117.2 (C-4a), 119.6 (C-5), 121.1 (C-4), 124.0 (C-4a), 125.9 (C-7), 128.0 (C-6), 139.0 (C-8a), 142.4 (C-9a), 159.5 (C-2). MS (70 eV): *m/z* (%) 211 (M^+^, 100), 196 (51), 168 (76), 139 (10), 86 (6). HRMS (EI): *m/z* [M^+^] calcd for C_14_H_13_NO: 211.0997; found: 211.0994.

*2,6-Dimethoxy-9H-carbazole* (**1i**). Following the procedure described for **1h**, with **5b** (0.100 g, 0.44 mmol), Pd(AcO)_2_ (0.0099 g, 0.044 mmol) and Cu(AcO)_2_ (0.198 g, 1.1 mmol), **1i** (0.086 g, 87%) was obtained as a white solid. *R*_f_ 0.55 (hexane/EtOAc, 7:3); mp 162–163 °C [Lit. [29] 163–164 °C]. IR (KBr): ν_max_ 3398, 1626, 1491, 1465, 1284, 1221, 1202, 1162, 1029, 820 cm^−1^. ^1^H-NMR (300 MHz, CDCl_3_): δ = 3.85 (s, 3H, C*H*_3_O), 3.86 (s, 3H, C*H*_3_O), 6.75 (dd, *J* = 8.4, 2.4 Hz, 1H, H-3), 6.92 (dd, *J* = 8.7, 2.5 Hz, 1H, H-7), 6.95 (d, *J* = 2.4 Hz, 1H, H-1), 7.31 (d, *J* = 8.7 Hz, 1H, H-8), 7.51 (d, *J* = 2.5 Hz, 1H, H-5), 7.89 (d, *J* = 8.4 Hz, 1H, H-4), 9.90 (br s, 1H, NH). ^13^C-NMR (75.4 MHz, CDCl_3_): δ = 53.9 (*C*H_3_O), 54.3 (*C*H_3_O), 93.3 (C-1), 101.2 (C-5), 106.6 (C-3), 110.0 (C-8), 112.0 (C-7), 115.9 (C-4a), 119.7 (C-4), 122.7 (C-4b), 133.8 (C-8a), 141.1 (C-9a), 152.8 (C-6), 158.0 (C-2). MS (70 eV): *m/z* (%) 227 (M^+^, 100), 212 (86), 184 (69), 169 (28), 141 (19), 114 (7). HRMS (EI): *m/z* [M^+^] calcd for C_14_H_13_NO_2_: 227.0946; found: 227.0951.

*2-Methoxy-7-methyl-9H-carbazole* (**1j**). Following the procedure described for **1h**, with **5c** (0.100 g, 0.47 mmol), Pd(AcO)_2_ (0.0105 g, 0.047 mmol) and Cu(AcO)_2_ (0.211 g, 1.17 mmol), **1j** (0.081 g, 82%) was obtained as a white solid. *R*_f_ 0.61 (hexane/EtOAc, 7:3); mp 162–163 °C [Lit. [68] 280 °C]. IR (film): ν_max_ 3399, 1654, 1047, 1025, 995, 827, 766 cm^−1^. ^1^H-NMR (500 MHz, DMSO-*d_6_*/acetone-*d_6_*, 3:7): δ = 2.46 (s, 3H, C*H*_3_), 3.85 (s, 3H, C*H*_3_O), 6.75 (dd, *J* = 8.5, 2.5 Hz, 1H, H-3), 6.95 (dd, *J* = 8.3, 1.0 Hz, 1H, H-6), 6.99 (d, *J* = 2.5 Hz, 1H, H-1), 7.25 (br s, 1H, H-8), 7.84 (d, *J* = 8.3 Hz, 1H, H-5), 7.88 (d, *J* = 8.5 Hz, 1H, H-4), 10.7 (br s, 1H, NH). ^13^C-NMR (125 MHz, DMSO-*d_6_*/acetone-*d_6_*, 3:7): δ = 21.4 (*C*H_3_), 54.9 (*C*H_3_O), 94.2 (C-1), 106.9 (C-3), 110.3 (C-8), 116.1 (C-4a), 118.4 (C-5), 119.6 (C-6), 119.9 (C-4), 120.2 (C-4b), 133.1 (C-7), 140.0 (C-8a), 140.8 (C-9a), 157.8 (C-2). MS (70 eV): *m/z* (%) 211 (M^+^, 100), 196 (58), 168 (66), 139 (10), 86 (6). HRMS (EI): *m/z* [M^+^] calcd for C_14_H_13_NO: 211.0997; found: 211.1000.

*2,7-Dimethoxy-9H-carbazole* (*Clausine V,* **1d**). Following the procedure described for **1h**, with **5d** (0.100 g, 0.44 mmol), Pd(AcO)_2_ (0.0099 g, 0.044 mmol) and Cu(AcO)_2_ (0.198 g, 1.10 mmol), **1d** (0.089 g, 90%) was obtained as a white solid. *R*_f_ 0.56 (hexane/EtOAc, 7:3); mp 229–230 °C [Lit. [22] 228–230 °C]. IR (KBr): ν_max_ 3382, 2927, 1608, 1575, 1502, 1457, 1322, 1265, 1233, 1160, 1118, 1026, 825, 805 cm^−1^. ^1^H-NMR (300 MHz, DMSO-*d_6_*/acetone-*d_6_*, 3:7): δ = 3.85 (s, 6H, 2C*H*_3_O), 6.75 (dd, *J* = 8.4, 2.4 Hz, 2H, H-3, H-6), 6.99 (d, *J* = 2.4 Hz, 2H, H-1, H-8), 7.85 (d, *J* = 8.4 Hz, 2H, H-4, H-5), 10.81 (br s, 1H, NH). ^13^C-NMR (75.4 MHz, DMSO-*d_6_*/acetone-*d_6_*, 3:7): δ = 54.9 (2*C*H_3_O), 94.6 (C-1, C8), 107.3 (C-3, C-6), 116.8 (C-4a, C-4b), 119.7 (C-4, C-5), 141.4 (C-8a, C-9a), 157.9 (C-2, C-7). MS (70 eV): *m/z* (%) 227 (M^+^, 77), 212 (100), 184 (42), 169 (54), 153 (13), 141 (27), 114 (5). HRMS (EI): *m/z* [M^+^] calcd for C_14_H_13_NO_2_: 227.0946; found: 227.0946.

*2,4,7-Trimethoxy-9H-carbazole* (**1k**). Following the procedure described for **1h**, with **5e** (0.101 g, 0.39 mmol), Pd(AcO)_2_ (0.0087 g, 0.039 mmol) and Cu(AcO)_2_ (0.175 g, 0.97 mmol), **1k** (0.092 g, 92%) was obtained as a white solid. *R*_f_ 0.20 (hexane/EtOAc, 7:3); mp 167–168 °C. IR (KBr): ν_max_ 3383, 1617, 1580, 1510, 1453, 1428, 1260, 1213, 1149, 1119, 1032, 803 cm^−1^. ^1^H-NMR (500 MHz, CDCl_3_/acetone-*d_6_*, 7:3): δ = 3.83 (s, 6H, 2C*H*_3_O), 3.98 (s, 3H, C*H*_3_O), 6.27 (d, *J* = 1.3 Hz, 1H, H-3), 6.46 (d, *J* = 1.3 Hz, 1H, H-1), 6.76 (dd, *J* = 8.5, 2.2 Hz, 1H, H-6), 6.82 (d, *J* = 2.2 Hz, 1H, H-8), 7.99 (d, *J* = 8.5 Hz, 1H, H-5), 9.32 (br s, 1H, NH). ^13^C-NMR (125 MHz, CDCl_3_/acetone-*d_6_*, 7:3): δ = 54.7 (*C*H_3_O), 54.9 (*C*H_3_O), 55.0 (*C*H_3_O), 86.7 (C-1), 90.2 (C-3), 94.0 (C-8), 106.0 (C-4a), 107.0 (C-6), 116.1 (C-4b), 121.7 (C-5), 139.7 (C-8a), 141.3 (C-9a), 155.2 (C-4), 156.8 (C-7), 158.7 (C-2). MS (70 eV): *m/z* (%) 257 (M^+^, 39), 247 (36), 230 (100), 219 (24), 214 (22), 190 (82), 176 (21), 160 (19), 117 (7). HRMS (EI): *m/z* [M^+^] calcd for C_15_H_15_NO_3_: 257.1052; found: 257.1052.

*7-Methoxy-9H-carbazole-3-carbaldehyde* *(Clauszoline-K)* (**1f**). A mixture of **1h **(0.030 g, 0.14 mmol) and DDQ (0.129 g, 0.57 mmol) in acetone/MeOH/H_2_O (1:1:1) (10 mL) was stirred at 25 °C for 45 min. The solvent was removed under vacuum and the residue purified by column chromatography over silica gel (10 g/g of crude, hexane/EtOAc, 8:2), to give **1f** (0.022 g, 70%) as a white solid. *R*_f_ 0.25 (hexane/EtOAc, 8:2); mp 184–185 °C [Lit. [32] 183–186 °C]. IR (KBr): ν_max_ 3296, 1670, 1604, 1570, 1479, 1322, 1237, 1160, 1026, 821 cm^−1^. ^1^H-NMR (500 MHz, DMSO-*d_6_*/CDCl_3_, 3:7): δ = 3.88 (s, 3H, C*H*_3_O), 6.84 (dd, *J* = 8.5, 2.0 Hz, 1H, H-6), 7.00 (d, *J* = 2.0 Hz, 1H, H-8), 7.51 (d, *J* = 8.0, 1H, H-1), 7.83 (dd, *J* = 8.0, 1.0 Hz, 1H, H-2), 7.96 (d, *J* = 8.5 Hz, 1H, H-5), 8.45 (s, 1H, H-4), 10.05 (s, 1H, CHO), 11.40 (br s, 1H, NH). ^13^C-NMR (125 MHz, DMSO-*d_6_*/CDCl_3_, 3:7): δ = 54.0 (*C*H_3_O), 93.8 (C-8), 107.6 (C-6), 109.5 (C-1), 115.0 (C-4b), 119.7 (C-5), 121.3 (C-4), 121.8 (C-4a), 124.1 (C-2), 126.9 (C-3), 140.8 (C-8a), 142.6 (C-9a), 158.0 (C-7), 190.2 (CHO). MS (70 eV): *m/z* (%) 225 (M^+^, 40), 210 (28), 180 (72), 167 (97), 160 (44), 146 (30), 130 (32), 115 (28), 77 (29), 51 (100). HRMS (EI): *m/z* [M^+^] calcd for C_14_H_11_NO_2_: 225.0790; found: 225.0796.

*Methyl 7-methoxy-9H-carbazole-3-carboxylate* *(Clauszoline-L, Clausine C)* (**1g**). A mixture of **1f**(0.200 g, 0.89 mmol), MnO_2_ (0.20 g, 2.3 mmol), and KCN (0.028 g, 0.43 mmol) in MeOH (10 mL) was stirred at 25 °C for 24 h. The solvent was removed under vacuum and the residue purified by column chromatography over silica gel (10 g/g of crude, hexane/EtOAc, 8:2), to give **1g** (0.22 g, 97%) as a white solid. *R*_f_ 0.29 (hexane/EtOAc, 8:2); mp 194–195 °C [Lit. [20] 195–197 °C; [32] 195 °C; [33] 194–195 °C]. IR (KBr): ν_max_ 3288, 1698, 1605, 1439, 1327, 1259, 1195, 1159, 1094, 815, 728 cm^−1^. ^1^H-NMR (500 MHz, acetone-*d_6_*): δ = 3.88 (s, 3H, C*H*_3_O), 3.91 (s, 3H, C*H*_3_O), 6.88 (dd, *J* = 8.5, 2.0 Hz, 1H, H-6), 7.08 (d, *J* = 2.0 Hz, 1H, H-8), 7.51 (d, *J* = 8.5, 1H, H-1), 7.99 (dd, *J* = 8.5, 1.5 Hz, 1H, H-2), 8.09 (d, *J* = 8.5 Hz, 1H, H-5), 8.69 (d, *J* = 1.5 Hz, 1H, H-4), 10.80 (br s, 1H, NH). ^13^C-NMR (125 MHz, acetone-*d_6_*): δ = 51.8 (*C*H_3_O), 55.7 (*C*H_3_O), 95.7 (C-8), 109.6 (C-6), 110.9 (C-1), 117.4 (C-4b), 121.6 (C-5), 121.9 (C-4a), 122.1 (C-4), 123.9 (C-3), 126.4 (C-2), 143.0 (C-8a), 144.0 (C-9a), 160.5 (C-2), 167.9 (*C*O_2_Me). MS (70 eV): *m/z* (%) 255 (M^+^, 100), 240 (22), 224 (44), 212 (61), 196 (38), 181 (33), 153 (67), 126 (15), 84 (20), 51 (21). HRMS (EI): *m/z* [M^+^] calcd for C_15_H_13_NO_3_: 255.0895; found: 255.0900.

*6-Methyl-2,3-dihydro-1H-carbazol-4(9H)-one* (**7a**). In a threaded ACE glass pressure tube with a sealed Teflon screw cap, under N_2_ atmosphere, a mixture of **4a** (0.10 g, 0.5 mmol) and Pd(AcO)_2_ (0.034 g, 0.15 mmol) in dry MeCN (2.5 mL) was stirred at 80 °C for 24 h. The solvent was removed under vacuum and the residue purified by column chromatography over silica gel (20 g/g of crude, hexane/EtOAc, 80:20), to give **7a** (0.077 g, 78%) as a white solid. *R*_f_ 0.15 (hexane/EtOAc, 1:1); mp 281–282 °C [Lit. [69] 280–282 °C]. IR (film): ν_max_ 3154, 2934, 1615, 1469, 1406, 1375, 1213, 1183, 1122, 1070, 1016, 797 cm^−1^. ^1^H-NMR (300 MHz, DMSO-*d_6_*): δ = 2.09 (qu, *J* = 6.3 Hz, 2H, H-2), 2.38 (s, 3H, C*H*_3_), 2.41 (t, *J* = 6.3 Hz, 2H, H-3), 2.93 (t, *J* = 6.3 Hz, 2H, H-1), 6.97 (br d, *J* = 8.1 Hz, 1H, H-7), 7.27 (d, *J* = 8.1 Hz, 1H, H-8), 7.77 (br s, 1H, H-5), 11.75 (br s, 1H, NH) ^13^C-NMR (75.4 MHz, DMSO-*d_6_*): δ = 21.2 (*C*H_3_), 22.7 (C-1), 23.4 (C-2), 37.8 (C-3), 111.1 (C-8), 111.4 (C-4a), 120.1 (C-5), 123.7 (C-7), 124.7 (C-4b), 130.2 (C-6), 134.1 (C-8a), 152.2 (C-9a), 192.8 (C-4). MS (70 eV): *m/z* (%) 199 (M^+^, 100), 198 (45), 183 (13), 170 (11), 154 (20), 128 (8), 91 (40).

*6-Methoxy-2,3-dihydro-1H-carbazol-4(9H)-one* (**7b**). Following the procedure described for **7a**, with **4b** (0.100 g, 0.46 mmol) and Pd(AcO)_2_ (0.0309 g, 0.138 mmol), **7b** (0.08 g, 80%) was obtained as a white solid. *R*_f_ 0.13 (hexane/EtOAc, 1:1); mp 252–253 °C [Lit. [70] 250–254 °C]. IR (KBr): ν_max_ 3416, 1578, 1482, 1459, 1259, 1217, 1175, 1031, 796, 780 cm^−1^. ^1^H-NMR (300 MHz, DMSO-*d_6_*): δ = 2.09 (qu, *J* = 6.3 Hz, 2H, H-2), 2.41 (t, *J* = 6.3 Hz, 2H, H-3), 2.92 (t, *J* = 6.3 Hz, 2H, H-1), 3.76 (s, 3H, C*H*_3_O), 6.77 (dd, *J* = 8.7, 2.7 Hz, 1H, H-7), 7.28 (d, *J* = 8.7 Hz, 1H, H-8), 7.45 (d, *J* = 2.7 Hz, 1H, H-5), 11.74 (br s, 1H, NH) ^13^C-NMR (75.4 MHz, DMSO-*d_6_*): δ = 22.8 (C-1), 23.4 (C-2), 37.7 (C-3), 55.2 (*C*H_3_O), 102.4 (C-5), 111.6 (C-7), 111.7 (C-4a), 112.2 (C-8), 125.2 (C-4b), 130.5 (C-8a), 152.3 (C-9a), 155.1 (C-6), 192.8 (C-4). MS (70 eV): *m/z* (%) 215 (M^+^, 2), 155 (37), 153 (100), 127 (12), 125 (35), 90 (23).

*6-Methyl-9H-carbazol-4-ol* (**8**). In a threaded ACE glass pressure tube with a sealed Teflon screw cap, under N_2_ atmosphere, a mixture of **7a** (0.20 g, 1.0 mmol) and Pd/C (10%) (0.060 g, 0.057 mmol) in dry MeOH (2.5 mL) was stirred at 270 °C for 48 h. The solvent was removed under vacuum and the residue purified by column chromatography over silica gel (20 g/g of crude, hexane/EtOAc, 80:20), to give **8** (0.168 g, 85%) as a white solid. *R*_f_ 0.30 (hexane/EtOAc, 7:3); mp 125–126 °C. IR (film): ν_max_ 3404, 1615, 1589, 1455, 1341, 1297, 1267, 1047, 803, 752, 724 cm^−1^. ^1^H-NMR (500 MHz, CDCl_3_): δ = 2.52 (s, 3H, C*H*_3_), 5.38 (br s, 1H, OH), 6.53 (d, *J* = 8.5 Hz, 1H, H-3), 6.95 (d, *J* = 8.5 Hz, 1H, H-1), 7.17–7.22 (m, 2H, H-2, H-7), 7.26 (t, *J* = 8.5 Hz, 1H, H-8), 7.89 (br s, 1H, NH), 8.06 (br s, 1H, H-5). ^13^C-NMR (125 MHz, CDCl_3_): δ = 21.4 (*C*H_3_), 103.3 (C-1), 104.9 (C-3), 109.7 (C-8), 111.6 (C-4a), 122.5 (C-4b), 122.7 (C-5), 126.3 (C-2 or C-7), 126.4 (C-7 or C-2), 129.0 (C-6), 137.0 (C-8a), 141.7 (C-9a), 151.8 (C-4). HRMS (EI): *m/z* [M^+^] calcd for C_13_H_11_NO: 197.0841; found: 197.0844.

*5-Methoxy-3-methyl-9H-carbazole* (*Glycoborine, Glycrophylamine,* **9**). A mixture of **8** (0.150 g, 0.76 mmol), MeI (0.216 g, 1.52 mmol) and K_2_CO_3_ (0.157 g, 1.14 mmol) in dry acetone (10 mL) was heated to reflux for 2 h. The solvent was removed under vacuum and the residue purified by column chromatography over silica gel (10 g/g of crude, hexane/EtOAc, 95:5), to give **9** (0.151 g, 94%) as a white solid. *R*_f_ 0.35 (hexane/EtOAc, 8:2); mp 133–134 °C [Lit. [33] 154–156 °C; [60] 132–134.6 °C; [61] 155–156 °C; [62] 135 °C]. IR (KBr): ν_max_ 3402, 1586, 1508, 1458, 1346, 1261, 1103, 804, 719 cm^−1^. ^1^H-NMR (500 MHz, CDCl_3_): δ = 2.52 (s, 3H, C*H*_3_), 4.06 (s, 3H, C*H*_3_O), 6.64 (d, *J* = 8.0 Hz, 1H, H-6), 6.97 (d, *J* = 8.0 Hz, 1H, H-8), 7.18 (dd, *J* = 8.0, 1.2 Hz, 1H, H-2), 7.24 (d, *J* = 8.0 Hz, 1H, H-1), 7.29 (t, *J* = 8.0 Hz, 1H, H-7), 8.00 (br s, 1H, NH), 8.11 (br s, 1H, H-4). ^13^C-NMR (125 MHz, CDCl_3_): δ = 21.4 (*C*H_3_), 55.3 (*C*H_3_O), 100.1 (C-6), 103.5 (C-8), 109.5 (C-1), 112.4 (C-4b), 122.8 (C-4a), 122.9 (C-4), 126.1 (C-2), 126.4 (C-7), 128.8 (C-3), 136.9 (C-9a), 141.2 (C-8a), 156.2 (C-5). HRMS (EI): *m/z* [M^+^] calcd for C_14_H_13_NO: 211.0997; found: 211.0995.

## 4. Conclusions

In this work, a short and efficient synthetic route for the construction of 2-, 5-, and 7-oxygenated carbazole alkaloids including natural clausine V (**1d**) is described. As the key steps, this approach includes a palladium(0)-catalyzed aromatization and a palladium(II)-catalyzed cyclization to provide the 2- and 7-oxygenated tricyclic carbazole framework. In the case of the natural 5-oxygenated carbazole glycoborine (glycrophylamine, **9**), the palladium-catalyzed sequence was inverted, with cyclization performed before aromatization. The preparation of natural carbazoles clauszoline-K (**1f**) and clauszoline-L (**1g**) was also carried out by transformation of carbazole **1h**. This methodology is currently being applied to the synthesis of diverse carbazoles, and the results will be reported in due course.
